# Analysis of interrater reliability in age assessment of minors: how does expertise influence the evaluation?

**DOI:** 10.1007/s00414-021-02707-8

**Published:** 2021-09-30

**Authors:** Lorenzo Franceschetti, Vera Gloria Merelli, Susanna Corona, Francesca Magli, Lidia Maggioni, Marco Cummaudo, Stefania Tritella, Danilo De Angelis, Cristina Cattaneo

**Affiliations:** 1 LABANOF, Laboratorio Di Antropologia E Odontologia Forense, Department of Biomedical Sciences for Health, University of Milan, Via Luigi Mangiagalli 37, 20133 Milan, Italy; 2 Forensic Medicine Unit, Department of Medical and Surgical Specialties, Radiological Sciences and Public Health, University of Brescia, Piazzale Spedali Civili, 1, 25123 Brescia, Italy; 3 Unit of Radiology, IRCCS Policlinico San Donato, Via Morandi 30, San Donato Milanese, 20097 Milan, Italy

**Keywords:** Age assessment, Clinical forensic medicine, Forensic anthropology, Unaccompanied minors, Interrater reliability

## Abstract

Nowadays, the clinical forensic medical management of migration flows comprises the age assessment of unaccompanied minors. The process of age estimation is a fundamental pillar for legally ensuring the minors’ rights and their protection needs. The procedure is complex and involves different phases and actors, from medical doctors to law enforcement officers. The present study aimed to investigate the performance of Greulich and Pyle, Demirjian, and Mincer methods when performed by raters both trained and without training. Also, the interrater reliability within groups of raters from different areas of expertise was evaluated. A total of 36 participants were enrolled for this study, divided in two groups according to their level of experience with age estimation methods. Each rater was asked to allocate stages and standards for age assessment, evaluating ten orthopantomograms and ten hand-wrist roentgenograms. The interrater reliability expressed through the Fleiss Kappa coefficient and the agreement with the reference standard were calculated. The results showed that none of the categories analyzed could reach a good interrater reliability (ĸ > 0.8) for both methods. The study results highlighted variation and disagreement in the interpretation of the sample among raters and in the subsequent stages and standards allocation. In conclusion, the results of this study highlight that expertise does influence the reliability of the most utilized methods of age estimation of living individuals and stress the importance of proper training and practice, which could greatly increase the accuracy of age assessments.

**Supplementary Information:**

The online version contains supplementary material available at 10.1007/s00414-021-02707-8.

## Introduction

In the global phenomenon of migration, age assessment has gained increasing importance as many countries are obliged to regulate, sort, and “place” the great numbers of individuals crossing their borders without identification documents. The management of migration flows comprises the identification and the protection of the most vulnerable among migrants, such as unaccompanied minors and asylum seekers. From a humanitarian perspective, the detention and treatment of minors as adults may have a negative impact on the individuals given their more susceptible condition to mental and emotional distress [1–3]. Today, age assessment not only does entail the legal guarantee of rights to minors, but also determines the criminal liability or conviction of adults involved in child pornography.

With regard to unaccompanied foreign minors, age estimation represents a fundamental step to ensure the fulfilment of their protection needs, and it is a complex procedure involving different phases and actors [4–9]. In the Italian context, the so-called Zampa Law (Law n.47/17) [10] provided comprehensive legislation aimed at filling existing gaps in the protection of unaccompanied minors arriving in Italy. Moreover, it introduced important provisions on age assessment procedure, which should only be performed when there is a reasonable doubt concerning a child’s age and by using the least invasive methods possible. Nevertheless, the law does not establish a specific trained professional figure responsible for age estimation. Different professionals can be in charge of the process, from medical doctors to law enforcement officers, often with interregional differences. In 2008, the *Study Group on Forensic Age Diagnostic* (AGFAD) provided recommendations for age estimation in the living [11], consisting in a three-step procedure which includes a physical examination (anthropometric data, assessment of sexual maturation and identification of potential age-relevant developmental disorders) and an evaluation of the dental status, along with X-ray examinations of the dentition, the left hand, and the clavicle. This latter method is used when the bones of the hand and wrist have completed their development. Indeed, the clavicle has an extended developmental period of its medial epiphysis, thereby providing accurate age estimates of young adults [12]. With a simple three-phase scoring system, the analysis of the medial clavicular epiphysis proved to be the least subjective, while retaining accuracy levels [13].

Although developed several decades ago, the Greulich and Pyle “Radiographic Atlas of Skeletal Development of the Hand and Wrist” [14] and the methods on dental development by Demirjian and colleagues [15] and Mincer and colleagues [16] are still commonly utilized in forensic practice and have been tested in different populations across the world [17]. However, at present, there are no studies that have examined how the level of expertise of the rater can influence the performance of such methods in the age assessment.

In this perspective, the present study has a twofold objective: On the one hand, it aims to investigate the performance of Greulich and Pyle, Demirjian, and Mincer methods when performed by raters trained on age assessment and raters without training; on the other hand, it aims to assess the reliability of agreement within six groups of raters belonging to different areas of expertise (forensic physicians, odontologists, anthropologists, radiologists, non-forensic physicians, and medical students).

## Materials and method

The sample sent to the participants consisted of ten orthopantomograms and ten hand-wrist roentgenograms belonging to twenty subjects from a database owned by the University Institute of Legal Medicine of Milan. The full database, obtained from the Sesto San Giovanni Hospital (Milan), consists of 385 orthopantomograms and 55 hand-wrist roentgenograms belonging to individuals ranging from 8 to 25 years. For the present study, the best radiographs in terms of image quality were chosen from subjects aged between 8 and 19 years (mean = 13.19 with SD = 3.11), with an equal ratio of boys and girls (1:1). No individuals presented any congenital or acquired malformation. An example of the questionnaire is provided in Supplementary Materials 1 (a 12-year-old male) and 2 (a 17-year-old female).

A total of 36 participants were enrolled for this study, divided in two main groups according to their level of experience with age estimation methods. The first group included six forensic physicians, six odontologists, six anthropologists, and six radiologists. Among them, four had sporadic experience in age assessment in the living (two anthropologists, a radiologist, and a forensic physician), whereas the remaining raters had only a basic training in age estimation during their academic education. The second group included six non-forensic physicians and six medical students without any training nor experience in age estimation methods.

Each rater received a survey comprising ten orthopantomograms and ten hand-wrist roentgenograms from twenty subjects, and they were asked to allocate stages and standards for age assessment of the individuals by applying the Demirjian method [15] and Greulich and Pyle atlas [14]. Whenever the highest Demirjian score was reached (98.4 for males and 100 for females), the Mincer method—based on wisdom teeth—was applied. This latter method explores the maturation stage of the third molar, which is typically the only tooth still in development during the young adult age [16]. The third molar is usually considered the most variable tooth of our dentition. The Mincer method is useful in age estimation in those cases in which the Demirjian score has reached its maximum discriminatory potential. A total of 740 determinations were performed.

The statistical analysis was conducted by calculating the interrater reliability expressed through the Fleiss Kappa coefficient [18]. Data obtained from surveys were recorded and entered in a digital data set and subsequently analyzed using Excel® software. In order to calculate the Fleiss Kappa coefficient, samples were organized according to age groups, e.g., 8–10 and 11–13, for a total of six categories. The Fleiss kappa was calculated for each group of raters (forensic physicians, odontologists, anthropologists, radiologists, non-forensic physicians, and medical students) and for groups according to the level of experience in age assessments (sporadic experience vs no experience). In addition, the allocations made by two professionals with over 10 years of experience in age assessment (an anthropologist and an odontologist) were used as the “reference standard” for the hand and wrist and dental age estimates respectively. These data were utilized in order to test the agreement between the “reference standard” and the allocations of stages and standards made by each of the other categories (e.g., radiologists vs “reference standard,” forensic physicians vs “reference standard,” anthropologists vs “reference standard”). For the dental age estimate, the agreement was analyzed not among estimated ages but among the stages assessed for each tooth of every radiograph, converting stages A–H to numbers (from 1 to 8).

## Results

### Interrater reliability

**Fig. 1:**
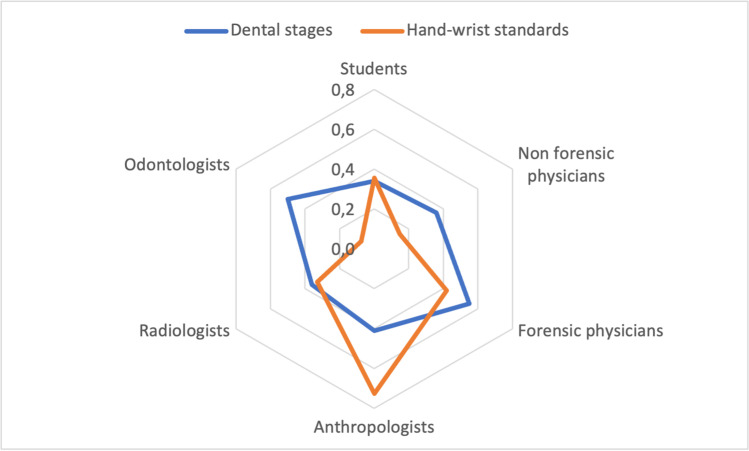
Fleiss kappa by professional category

The results of the statistical analysis for the interrater reliability are shown in Fig. 1. Overall, the highest Fleiss Kappa for the dental age estimates was obtained by forensic physicians (0.54), followed by odontologists (0.49), anthropologists (0.41), radiologists (0.36), non-forensic physicians (0.35), and medical students (0.34). Concerning the Greulich and Pyle atlas (hand-wrist), the highest interrater reliability was attained by anthropologists (0.72), followed by forensic physicians (0.41), radiologists (0.33), medical students (0.32), non-forensic physicians (0.14), and odontologists (0.07).

### Influence of the expertise of the participants

**Fig. 2:**
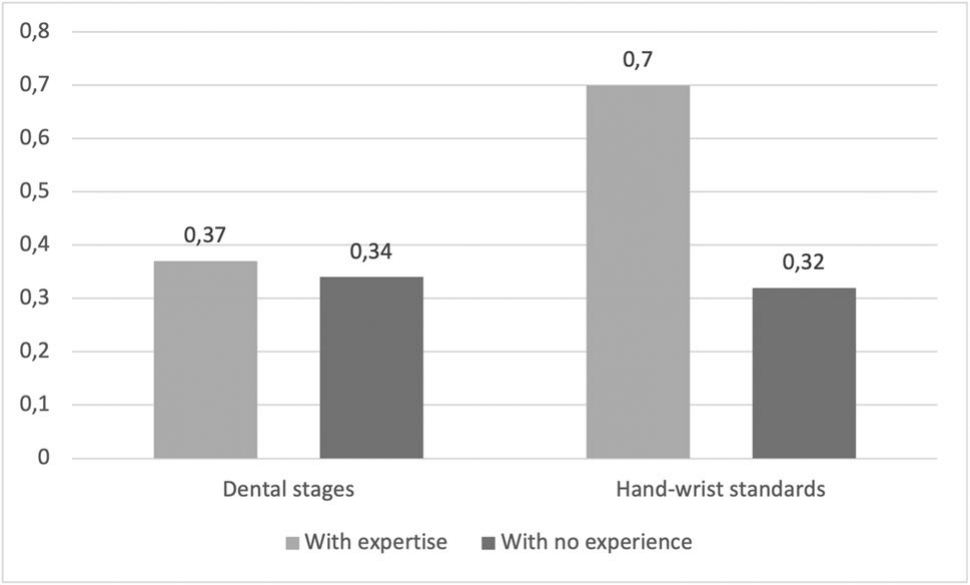
Fleiss kappa by experience

As illustrated in Fig. 2, according to the expertise, the “expert group” achieved a higher interrater reliability compared to the group without experience. In fact, the analysis of Fleiss kappa coefficient for raters with sporadic or continuous experience in age estimation resulted in a Fleiss kappa of 0.37 for the dental methods and 0.70 for the hand-wrist method. The raters without experience obtained a Fleiss kappa of 0.34 for the dental methods and 0.32 for the hand-wrist method.

### Comparison with the “reference standard”

**Fig. 3:**
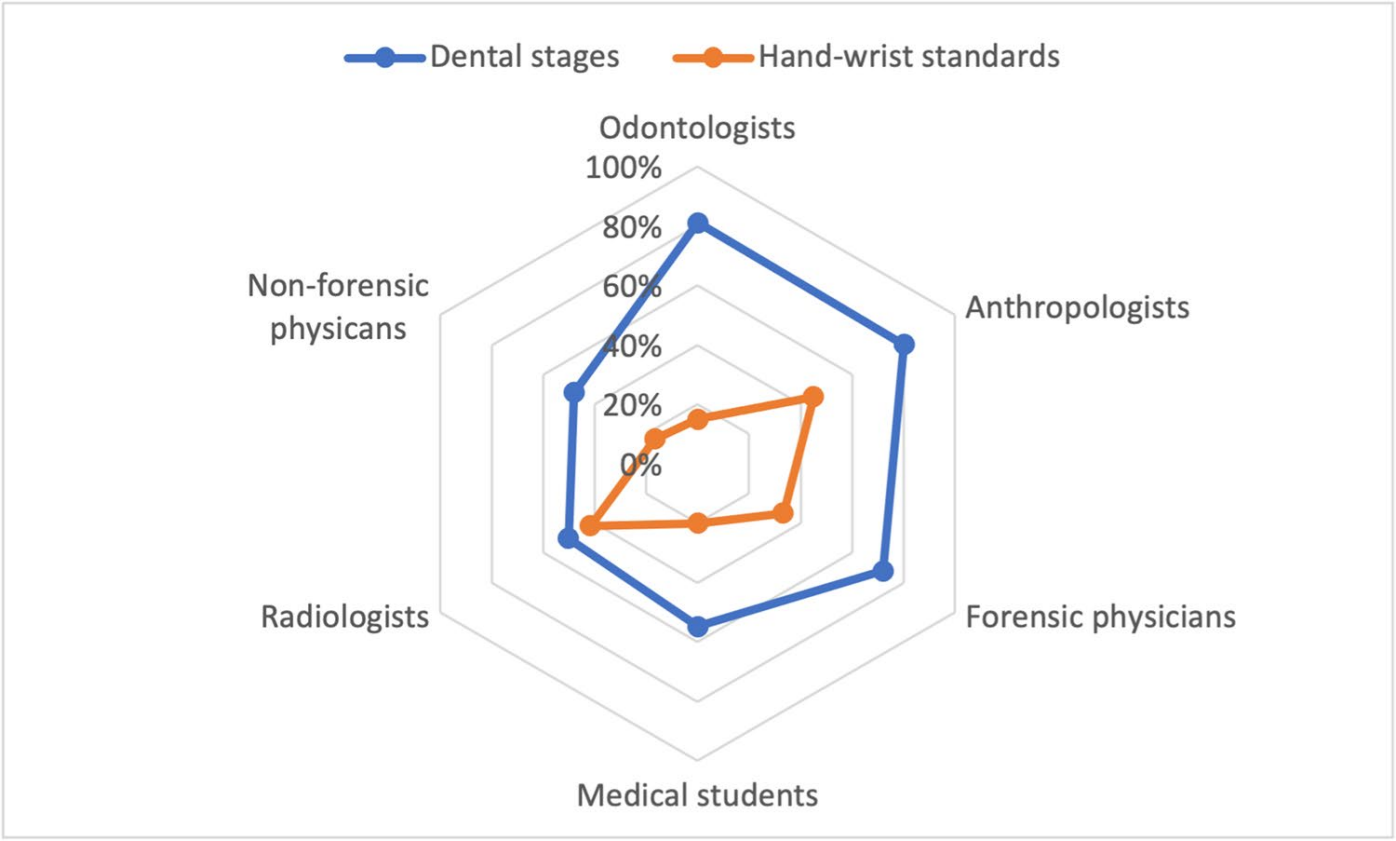
Percentages of agreement between each category of raters with the reference standard

The comparison between each category of raters with the “reference standard” (Fig. 3) have shown that the highest rate of agreement for dental methods was obtained by forensic anthropologists (81%), followed by odontologists (76%), forensic physicians (63%), radiologists (38%), non-forensic physicians (30%), and medical students (26%). With regard to Greulich and Pyle atlas, the highest rate of agreement was achieved by forensic anthropologists (45%), followed by radiologists (30%), forensic physicians and medical students (20%), non-forensic physicians (16%) and odontologists (15%).

## Discussion

Age assessment of the living has become an important pillar of the forensic practice [17]. One of the main problems regarding age estimation is the impossibility to get an accurate and definitive result [11, 19–25]. The main reason for this is the intrinsic biological variability of development among different individuals, depending on both genetic and environmental conditions. Each child has a specific and individual pattern of growth that can vary widely depending on the socio-economical, nutritional, and social status of the child as well as his/her level of physical activity [26–32].

Despite a number of methods for age estimation of living individuals having been developed and tested on several populations across the world [17], it is still unclear whether these methods can be applied successfully by raters belonging to different areas of expertise [33–36]. Moreover, no study has yet investigated how the rater’s experience in applying those methods can influence the reliability of the age estimate. Although population studies are needed, studies on intrinsic method precision may be even more meaningful than testing on continuously changing populations, especially with the current increase in migration flows. In fact, a study by Thevissen and colleagues [6] showed that country-specific databases hardly increased the mean absolute difference. Consistently, a meta-analysis of published data from retrospective studies of dental maturity from eight countries showed no major statistical differences in the timing of tooth formation stages [34]. These studies were both on dental development, which is known to be less affected by environmental factors than skeletal development. Even regarding skeletal development, however, today’s populations are far from constant and uniform. A sample of a given population at a certain time is not necessarily representative of the same population at another time, as a period of time has passed characterized by emigration or immigration flows. Moreover, as the interval of time extends, environmental conditions such as nutrition habits or basic quality of life also could also have changed.

In this regard, the interobserver reliability of the most commonly utilized methods for skeletal and dental age estimation was evaluated. This was done on the grounds that the replication of results means that a method and the results obtained from it are valid. Indeed, the calculation of the interobserver error is critical for an objective comparison of different methods, independently of the sample type. The Fleiss kappa coefficient represents the most popular index to evaluate interrater agreement: In particular, a ĸ value > 0.8 is generally considered the minimum satisfactory result [18, 37]. Although Cohen’s Kappa coefficient is also frequently used in forensic anthropology, it is not without problems that can yield misleading conclusions under certain conditions [38, 39].

The present study demonstrated how none of the categories analyzed (forensic physicians, forensic anthropologists, odontologists, radiologists, non-forensic physicians, and medicine students) could reach a good interrater reliability (ĸ > 0.8) for both dental and skeletal methods. The highest kappa coefficient was reached by forensic anthropologists (0.73) for the Greulich and Pyle method and by forensic physicians for dental methods (0.55). Overall, the dental methods obtained a higher degree of congruence compared to the Greulich and Pyle atlas. It should be considered that with the exception of the six experts, all the other raters received only a basic training (or no training at all) on skeletal and dental age estimation methods during their academic education. None of them has ever had a direct experience in age estimation in the living. Age assessment requires a specific set of skills (both theoretical and practical) that cannot be fully acquired during the work experience of the different medical and non-medical professionals who can deal with age estimation, such as those considered in this study. In this respect, as expected, the “expert group” achieved a higher interrater reliability compared to the group without experience (non-forensic physicians and medical students). It is interesting to note that higher interrater reliability could be achieved by trained personnel using the hand/wrist standards and that the dental stage method was overall less reliable and less sensitive to training. This may be due to the fact dental methods consist of the sum of different stages on many teeth and therefore could be subjected to more variability, compared to the qualitative and more general hand/wrist methods which are less structured. However, the possible disagreement between raters concerns mainly the second and thirds molars, hence reducing the number of variables observed in the dentition making it more comparable to the hand-wrist assessment.

Consequently, understanding the characteristics of dental and skeletal indicators of development and the standardization of their description remains a crucial topic to address for correct age estimation, documentation of comparable data, and accurate assessment. It is likely that specific training in these methods will increase the accuracy rates and reduce the variation observed among participants, regardless of their field of specialization or experience.

In the European context, where a proper ascertainment of age in minors is frequently badly managed [9], this is extremely important. Practical guidelines for age assessment of minors [40, 41] recommend adopting a multidisciplinary holistic approach [2]. In Italy, the daily practice does not include the existence of specifically trained personnel in hospitals nor do most physicians or policymakers know about the above-mentioned standards, apart from the smaller forensic community which is not present in most hospitals. Radiological methods are frequently left as a last resort, even if they are more quantifiable and reliable than neuropsychiatric and psychological evaluations [42]. Professionals with very different types of training may perform the age estimate, from social workers to psychologists, to medical doctors and law enforcement officers, with consequent paradoxes and poor administration of the rights of minors.

Limitations of this study include the limited number of radiographs analyzed. The number can be considered sufficient for the analysis of the interrater reliability (which is the main focus of this study), whereas, in order to test the accuracy of the age assessments when compared to the real ages, a larger sample would have be more adequate. However, this can be considered a pilot study, and further studies must be conducted on larger samples. In addition, although the present research showed how expertise plays a role in the method’s applicability, it refers to stage allocation for teeth and hand/wrist, and not to the specific age estimate. Further considerations about age estimations based on the stages and standards can be made only following the examination of a larger population. Moreover, this investigation involved only Italian experts in different areas, but the recruitment of more experts, including those of the international community, is likely to take place in subsequent research. Finally, it might be interesting to evaluate how the quality of radiographic images can influence the interrater reliability of both skeletal and dental methods.

In conclusion, the results of this study highlight that expertise does have an effect on the reliability of the most commonly utilized methods of age estimation of living individuals and show the importance of proper training and practice, which could greatly increase the accuracy of age assessments.

## Supplementary Information

Below is the link to the electronic supplementary material.

Supplementary Fig. 1 An example of orthopantomogram sent to the participants (a 12-year-old male). (PNG 294 KB)

High Resolution Image (TIFF 243 KB)

Supplementary Fig. 2 An example of hand-wrist roentgenogram sent to the participants (a 17-year-old female). (PNG 500 KB)

High Resolution Image (TIFF 279 KB)

## Data Availability

The authors confirm that the data supporting the findings of this study are available within the article.
