# Supplementary figures and images for: Analysis of interrater reliability in age assessment of minors: how does expertise influence the evaluation?
Source: Int J Legal Med. 2021 Sep 30;136(1):279–85. doi: 10.1007/s00414-021-02707-8 (PMC8813704; doi:10.1007/s00414-021-02707-8)

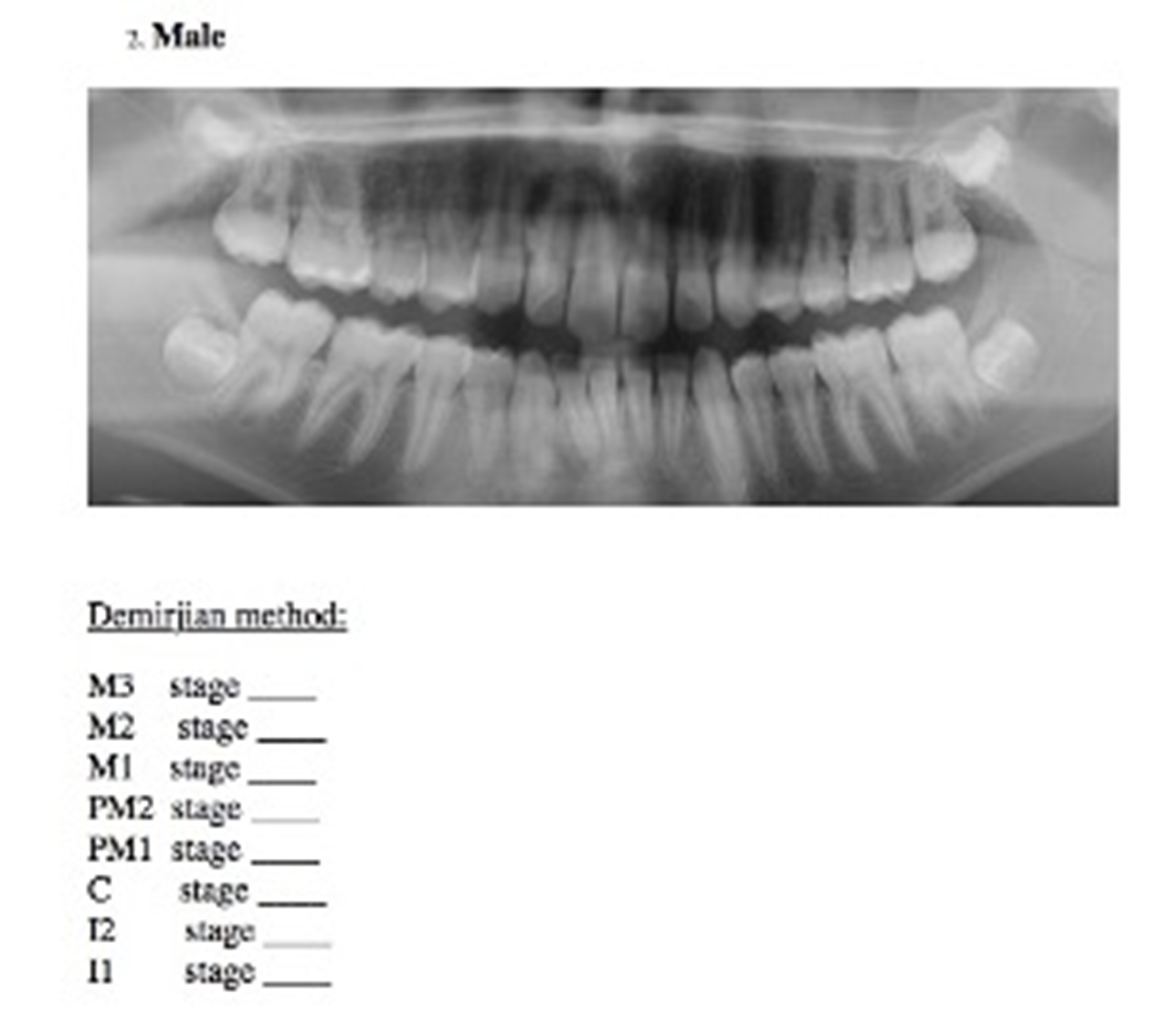

Supplement: Supplementary file 1 — An example of orthopantomogram sent to the participants (a 12-year-old male). (PNG 294 KB) [file 414_2021_2707_Fig4_ESM.png]

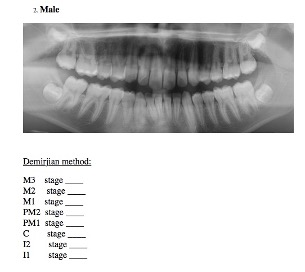

Supplement: Supplementary file 2 — High Resolution Image (TIFF 243 KB) [file 414_2021_2707_MOESM1_ESM.tiff]

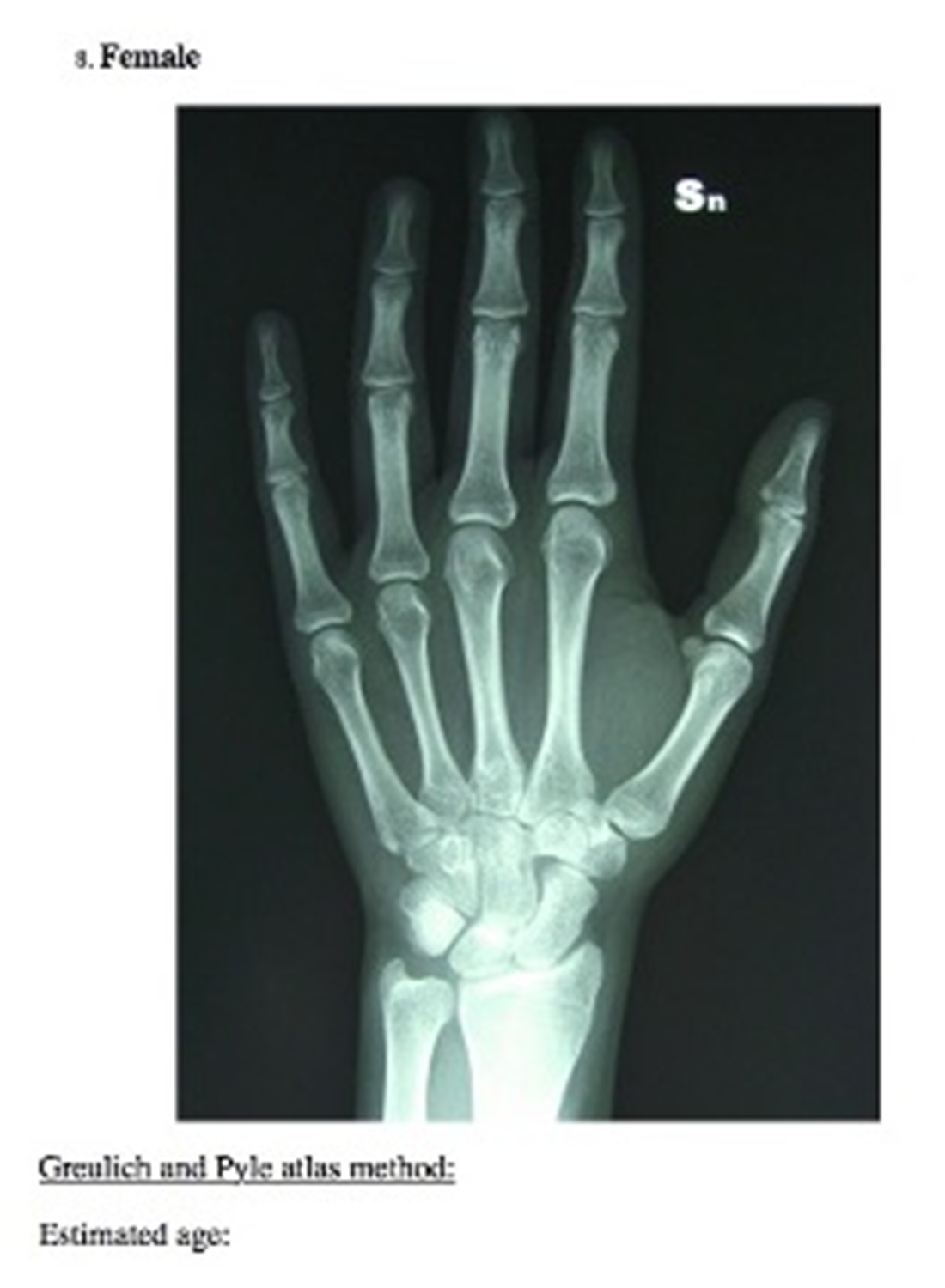

Supplement: Supplementary file 3 — An example of hand-wrist roentgenogram sent to the participants (a 17-year-old female). (PNG 500 KB) [file 414_2021_2707_Fig5_ESM.png]

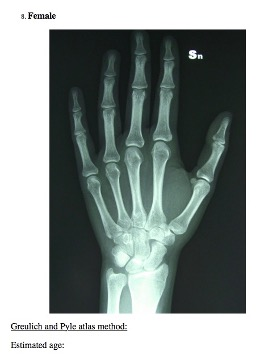

Supplement: Supplementary file 4 — High Resolution Image (TIFF 279 KB) [file 414_2021_2707_MOESM2_ESM.tiff]
